# The Agr communication system provides a benefit to the populations of *Listeria monocytogenes* in soil

**DOI:** 10.3389/fcimb.2014.00160

**Published:** 2014-11-06

**Authors:** Anne-Laure Vivant, Dominique Garmyn, Laurent Gal, Pascal Piveteau

**Affiliations:** ^1^Unités Mixtes de Recherche1347 Agroécologie, Université de BourgogneDijon, France; ^2^Institut National de la Recherche Agronomique, Unités Mixtes de Recherche1347 AgroécologieDijon, France; ^3^AgroSup Dijon, Unités Mixtes de Recherche1347 AgroécologieDijon, France

**Keywords:** Agr system, cell communication, competitiveness, fitness, *Listeria monocytogenes*, soil, biotic interaction

## Abstract

In this study, we investigated whether the Agr communication system of the pathogenic bacterium *Listeria monocytogenes* was involved in adaptation and competitiveness in soil. Alteration of the ability to communicate, either by deletion of the gene coding the response regulator AgrA (response-negative mutant) or the signal pro-peptide AgrD (signal-negative mutant), did not affect population dynamics in soil that had been sterilized but survival was altered in biotic soil suggesting that the Agr system of *L. monocytogenes* was involved to face the complex soil biotic environment. This was confirmed by a set of co-incubation experiments. The fitness of the response-negative mutant was lower either in the presence or absence of the parental strain but the fitness of the signal-negative mutant depended on the strain with which it was co-incubated. The survival of the signal-negative mutant was higher when co-cultured with the parental strain than when co-cultured with the response-negative mutant. These results showed that the ability to respond to Agr communication provided a benefit to listerial cells to compete. These results might also indicate that in soil, the Agr system controls private goods rather than public goods.

## Introduction

For the last few decades, communication between bacteria has raised a growing interest. Cell-to-cell communication is based on the synthesis, the diffusion between cells and the perception of signal molecules. The perception of these molecules in the cell's extracellular environment induces the regulation of transcription and eventually adjustment of the physiology of the cell to its surrounding environmental conditions. Various communication systems have been described in the prokaryotic world. They differ according to the type of signal molecules and the machinery used to integrate the signal. To date, the communication systems most studied involve cyclic peptides (AIP), acyl-homoserine lactones (acyl-HSL) or auto-inducer-2 (AI-2) as signal molecules (Miller and Bassler, 2001; Reading and Sperandio, 2006; Atkinson and Williams, 2009).

Several social traits are regulated through cell-to-cell communication. Adhesion, biofilm formation and mobility require functional communication systems in several bacterial species (Labbate et al., 2004; Yarwood et al., 2004; Sturme et al., 2005; Rieu et al., 2007; Boles and Horswill, 2008; Fujii et al., 2008; Jayaraman and Wood, 2008; Riedel et al., 2009; Ray and Visick, 2012; Bowden et al., 2013). Public goods are exo-products as for example, virulence factors, surfactants or antibiotics produced and secreted by bacterial populations. Their production is usually under the control of the spatial distribution and density of cells and is dependent on the characteristics of mass transfer in the environment. For example, in *Staphylococcus aureus* (Morfeldt et al., 1995; Novick and Geisinger, 2008), *Enterococcus faecalis* (Qin et al., 2001; Nakayama et al., 2006), *Clostridium perfringens* (Vidal et al., 2011; Chen and McClane, 2012), *Pseudomonas aeruginosa* (Passador et al., 1993; Pearson et al., 1997), and *Listeria monocytogenes* (Autret et al., 2003; Riedel et al., 2009), communication systems control the secretion of the virulence factors required for the onset of infection. Moreover, survival mechanisms, such as sporulation, granulose formation, and antibiotic production are also controlled by communication systems in *Clostridium acetobutylicum* (Steiner et al., 2012), *Pseudomonas chlororaphis* (Morohoshi et al., 2013), and *Bacillus subtilis* (Comella and Grossman, 2005). These communication-dependent coordinated behaviors are examples of cooperation in the microbial world (Keller and Surette, 2006; Diggle et al., 2007). Such a social trait is vulnerable to exploitation by cheaters, these individuals that do not cooperate but gain the benefit from others cooperating (Velicer, 2003). Cheaters are individuals unable either to respond to the signal or to synthesize it. Cheaters have been isolated from populations of clinical and environmental *P. aeruginosa* (Salunkhe et al., 2005; Heurlier et al., 2006). Saving the cost of the production of the signal molecules, of their detection or production of exo-products (Diggle et al., 2007) may give cheaters an advantage and may decrease the value of cooperation (West et al., 2002; Rainey and Rainey, 2003). Experimentally, under controlled environments where access to public goods is required for growth, cheaters are fitter than individuals that cooperate (Rainey and Rainey, 2003; Diggle et al., 2007). Assessing the value of cooperation in natural settings is required in order to understand why communication and cooperation behaviors have been conserved so far in bacteria.

We tackled this issue with the bacterial model *L. monocytogenes* as this food-borne pathogen is ubiquitous in nature. It has been isolated from water systems (De Luca et al., 1998; Paillard et al., 2005; Lyautey et al., 2007), vegetation (Welshimer, 1968; Beuchat, 1996), farms (Nightingale et al., 2004; Fox et al., 2009; Latorre et al., 2010; Strawn et al., 2013), food industries (Goulet et al., 1998; Garrido et al., 2009; Serio et al., 2011), and feces of animals (Fenlon, 1985; Iida et al., 1991). It is also found in soil (Welshimer, 1960; Weis and Seeliger, 1975; Locatelli et al., 2013a; Vivant et al., 2013a). A communication system has been characterized in this organism. It is the Agr system that regulates adhesion, biofilm formation (Rieu et al., 2007; Riedel et al., 2009) and infection of mammalian hosts (Autret et al., 2003; Riedel et al., 2009). Four genes, *agrBDCA*, code the proteins required for Agr communication (Autret et al., 2003; Garmyn et al., 2009). Among them, *agrD* codes the propeptide AgrD processed into a mature autoinducing peptide (AIP) by AgrB; AgrA, the transcriptional regulator of the two component system AgrC/AgrA, is the response component of the system. Detection of AIP by the sensor AgrC triggers activation of AgrA. In order to investigate whether or not cooperation through communication provided an advantage to populations of *L. monocytogenes* in complex, natural environments, we compared the behavior of two communication mutants, a signal-negative mutant Δ agrD unable to produce AIP but equipped to sense and respond to AIP, and a response-negative mutant Δ agrA unable to respond to extracellular signal, to the behavior of the parental strain following inoculation in soil.

## Materials and methods

### Bacterial strains and culture media

Rifampicin resistant strains were used in this study. The parental strain *L. monocytogenes* L9 is derived from *L. monocytogenes* EGD-e (Lemunier et al., 2005). Rifampicin resistant isogenic mutants *L. monocytogenes* DG125A6 (this study) and *L. monocytogenes* DG119D9 (this study), respectively are *ΔagrA* and *ΔagrD* in-frame deletion mutants (Rieu et al., 2007). Rifampicin resistant strains were isolated on Polymyxin-Acriflavin-Lithium-Chloride-Ceftazidime-Aesculin-Mannitol agar (PALCAM; AES chemunex, Bruz, France) supplemented with 200 μ g.ml^−1^ rifampicine (Sigma-Aldrich, Saint Quentin Fallavier, France) according to Lemunier et al. (2005). For each strain, spontaneous Rif^R^ mutants were selected by comparing growth rates during planktonic growth and the ability to grow as biofilm in tryptone soy broth (TSB; AES chemunex, Bruz, France) at 25°C without shaking. *L. monocytogenes* DG125A6 was used as a response-negative mutant and *L. monocytogenes* DG119D9 as a signal-negative mutant.

A working stock stored at −80°C was used throughout the study. Strains were grown statically at 25°C for 16 h in 5 ml of TSB. Three independent inocula were prepared by inoculating 10 ml of TSB (1% v/v) and incubating statically at 25°C to an O.D_600nm_ of 0.4. The cultures were then centrifuged at 8000 g for 5 min at room temperature and pellets were suspended in NaCl (0.85%). Cultures were adjusted to a concentration of 2.10^8^ CFU/ml.

### Soil samples and soil microcosms preparation

Soil was sampled in a pasture located in Burgundy, France. This sampling site belongs to a country-wide soil sampling network (RMQS) based on a 16 × 16 km systematic grid covering the whole of France (Arrouays et al., 2002). Twenty-five individual core samples of topsoil (0–30 cm) were taken using a sampling design within an area of 20 × 20 m. The core samples were then mixed to obtain a composite sample. The soil sample was then sieved to 5 mm and stored at 4°C. Aliquots of the soil were heat sterilized three times (120°C, 20 min) with a period of 24 h between each autoclave treatment. Fifty g of sterilized and non-sterilized soil were packed in triplicate to constitute sterilized and biotic soil microcosms. Soil's attributes such as location, composition, chemistry, and land use are stored in the DONESOL database (Grolleau et al., 2004). Briefly, it is a clay soil with neutral pH. Organic carbon and nitrogen content were respectively 35.3 and 3.9 g.kg^−1^.

### Soil microcosm inoculation with single strain and co-inoculation

Single strain cultivation in biotic and sterilized soil were performed by inoculating a single strain, either *L. monocytogenes* L9, *L. monocytogenes* DG125A6 or *L. monocytogenes* DG119D9, at a concentration of 2.10^6^ CFU/g in 50 g soil microcosms. Microcosms were also co-inoculated with appropriate mixtures from individual cultures to a final ratio of 50:50 (2.10^6^:2.10^6^ CFU/g). The following listerial mixtures were tested: *L. monocytogenes* L9/*L. monocytogenes* DG125A6, *L. monocytogenes* L9/*L. monocytogenes* DG119D9, and, *L. monocytogenes* DG125A6/*L. monocytogenes* DG119D9. Experiments were prepared in triplicates. All inoculated and co-inoculated microcosms were incubated at 25°C in the dark.

### Enumeration and determination of listerial populations dynamics

For single-cultures, listerial populations were enumerated by serial plating on Polymyxin-Acriflavin-Lithium-Chloride-Ceftazidime-Aesculin-Mannitol agar (PALCAM; AES Chemunex, Bruz, France) supplemented with 100 μg.l^−1^ cycloheximide and 100 μg.l^−1^ rifampicin (Sigma-Aldrich, Saint Quentin Fallavier, France) immediately after inoculation and periodically over a 14-days period for microcosms or over a 48-h period for extracts.

In microcosms inoculated with 50/50 mixtures, the total number of listerial cells was enumerated as described above. The proportion of each of the two strains was determined by strain-specific PCR amplification (described below) from up to 96 colonies collected from the supplemented PALCAM plates.

### PCR amplification

DNA template was prepared by transferring each colony in 200 μl of water. Three sets of strain-specific primers were designed to discriminate co-inoculated strains. Two PCR reactions with two of the primer sets were required to discriminate co-inoculated strains. The sequences of the strain-specific primer sets and the genotype targeted are shown Table 1. PCR amplification was carried out in a final volume of 20 μl containing 2.5 μl of DNA template, 1 μl of dimethyl sulfoxide (DMSO, Sigma-Aldrich, Saint Quentin Fallavier, France), 2 μl of 10X PCR buffer with MgCl_2_, 0.16 μl of dNTP mix (25 mM), 1.0 U of Taq polymerase (MP Bio, Illkirch Graffenstaden, France), and a final concentration of 0.6 μM of each primer. The following conditions were specifically determined and used: 95°C for 10 min, 30 cycles of 15 sec at 95°C, 50°C for 1 min and 72°C for 2 min, followed by 7 min at 72°C.

**Table 1:** **Sequences of the strain-specific primer sets and genotypes targeted**.

**Primer set**	**Oligonucleotide sequence 5′ → **3**′**	**Genotype targeted *L. monocytogenes:***		
		**L9**	**DG125A6**	**DG119D9**
C10	CTTCAAACCCGGCATATCAT	+	+	+
C11	GGAATGTTGGCGAATTTGTT			
A19	AATCCATGGTACCGGTTTTTATTTGT	+	−	+
A20	CTCGAGTAAACTCAAGCTTTTAATTA			
B7	AGCTAGCTGTCATGAAGTTTGCTCTCG	+	+	−
D2	AAGAATCCGCAACTTTCATGG			

*+ amplification, − no amplification*.

### Competitive index determination

For each of the three replicates, the competitive Index (CI) was calculated as follows:

$${\text{CI}}_{\text{tx}}\;=\;({\left({\text{CFU}}_{\text{mutant}}{\text{/CFU}}_{\text{parental}}\right)}_{\text{tx}}\text{/}{\left({\text{CFU}}_{\text{mutant}}{\text{/CFU}}_{\text{parental}}\right)}_{\text{t0}})$$

Where CI_tx_ is the competitive index at time tx (*x* = 2 days, 4 days, 7 days or 14 days), CFU_mutant_ and CFU_parental_ are the number of Colony Forming Units per gram of soil of the mutant and the parental strains, respectively, at time tx and at time t0. A CI score of 1 indicates no fitness difference. A similar calculation was realized for co-cultured listerial mutants.

### Statistical analysis

Patterns of survival of listerial populations were compared by repeated-measures analysis of variance (repeated-measures ANOVA) in both sterilized and biotic microcosms. To estimate whether or not the CI evolved over time, thus to determine whether a strain had a better ability to compete in soil, repeated-measures analysis of variance (repeated-measures ANOVA) was performed.

## Results and discussion

### Dynamics of listerial populations in soil microcosms

**Figure 1:**
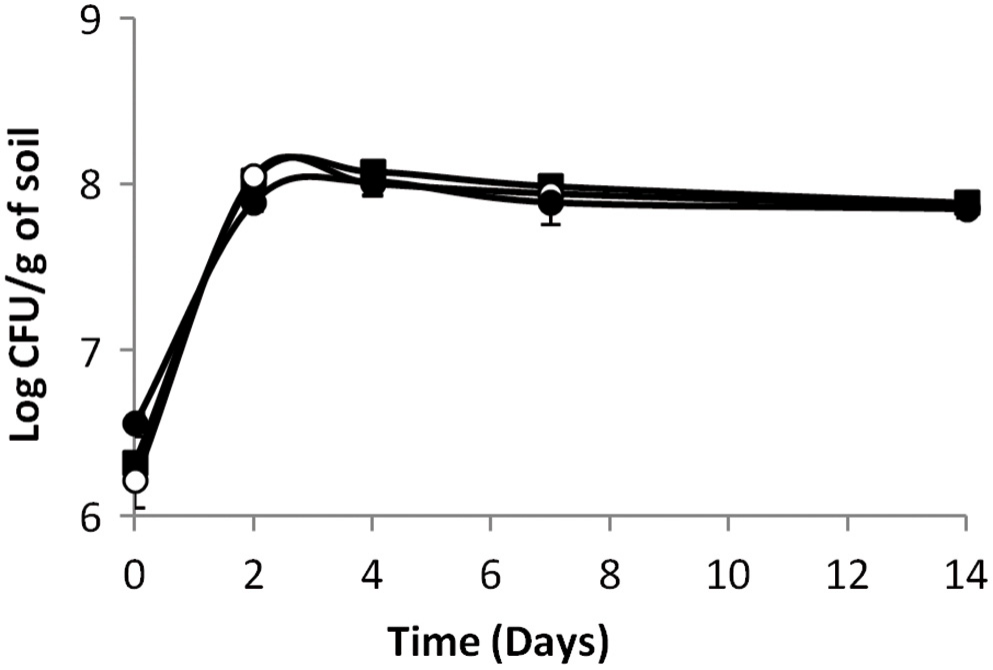
**Growth kinetics of *L. monocytogenes* (◼) parental strain, (⚫) signal- and (○) response-negative mutants in sterilized soil microcosms**. Error bars represent the standard deviation from three replicate samples value.

In sterilized soil microcosms, the population of the parental strain *L. monocytogenes* L9 increased of over 2 log within the first 2 days of incubation and the population remained stable until the end of the experiment (Figure 1). Inactivation of the Agr system did not affect the dynamics of the mutants' population and no significant differences were observed between growth profiles of the parental strain, the signal-negative *ΔagrD* mutant and the response-negative *ΔagrA* mutant. Similar results were collected during growth in sterilized soil extracts (data not shown). These results confirm previous reports on the ability of *L. monocytogenes* to multiply in sterilized soil (Dowe et al., 1997; Moshtaghi et al., 2009; McLaughlin et al., 2011; Piveteau et al., 2011). Moreover, our results suggest that the ability to produce AIP and to respond to the signal is not indispensable for growth of *L. monocytogenes* in this specific environment.

**Figure 2:**
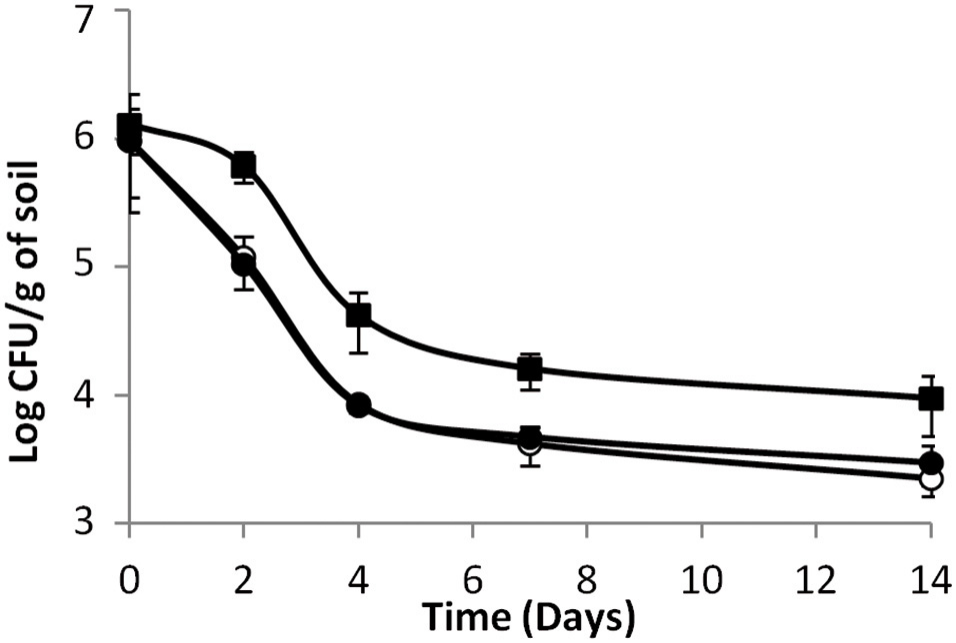
***L. monocytogenes* (◼) parental strain, (⚫) signal- and (○) response-negative mutants survival in biotic soil microcosms**. Error bars represent the standard deviation from three replicate samples value.

When indigenous microflora was not inactivated, in biotic soil microcosms, results were different (Figure 2). First of all, no growth was observed. The population of the parental strain was stable during the first 2 days of incubation thereafter the population declined throughout the duration of the experiment. Furthermore, the behavior of the mutants was significantly different. Indeed, the population of the two mutants declined sharply within the first 2 days of incubation and it was over 1 log lower than that of the parental strain from day 2 to the end of the experiment (*P* < 0.05). Differences between mutants were not significant. The results point out to the role of endogenous microbial communities in limiting implantation of *L. monocytogenes* in soil. Indeed, inactivation of telluric communities lifts inhibition (Dowe et al., 1997; Locatelli et al., 2013b; Vivant et al., 2013b). Moreover, microbial diversity is critical regarding the ability of soil microbial communities to limit invasion by *L. monocytogenes* (Vivant et al., 2013b). Our data strongly suggest that the activity of the Agr communication system is required for optimal survival of *L. monocytogenes* in soil. This suggests that the production of signal molecules and/or AgrA-mediated regulation improves the fitness of the populations of *L. monocytogenes* in soil. Moreover, production of private or public goods could be involved. In order to figure out if signal sensing in one hand or public goods production in the other hand underpinned the fitness advantage of the parental strain, we followed the fate of populations of the signal-negative and response-negative mutants during co-incubation with the parental strain in soil microcosms.

### Comparison of the fitness of the mutants and parental strains in soil microcosms

To determine if alteration of the Agr communication system affected fitness in soil, we measured the survival of the parental strain and of both mutants in sterilized or biotic soil depending on whether they had been cultured with the parental strain, a mutant or as single listerial population. Moreover, Competitive Indexes (CI) of co-cultured listerial strains over a 14-days period in soil microcosms were calculated.

**Figure 3:**
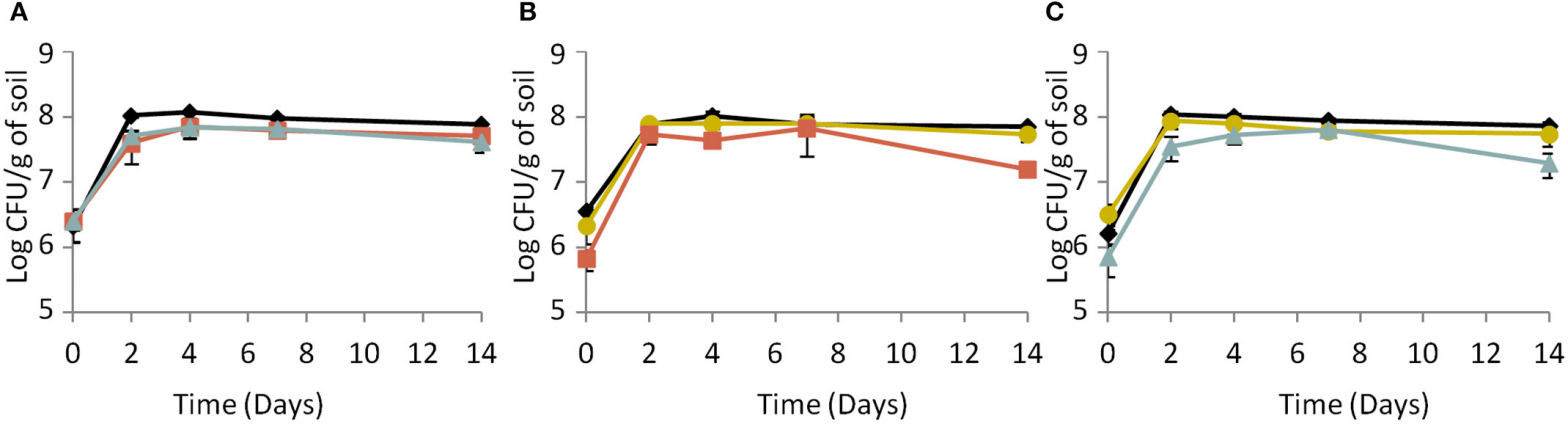
**Dynamics of (A) the parental strain, (B) the response-negative mutant and (C) the signal-negative mutant populations in sterilized soil microcosms**. (♦) Single culture, (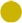) co-culture with the parental strain, (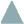) co-culture with the response-negative mutant, (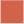) co-culture with the signal-negative mutant. Error bars represent the standard deviation from three replicate samples value.

As shown in Figure 3, in sterilized soil microcosms, colonization profiles were similar for all strains whether they had been cultured as a single strain or with a partner. Moreover, variations of the CI were not significant (ANOVA, *P* > 0.05) (Table 2). This is consistent with the results described above and confirms that in sterilized soil, in the absence of biotic pressure, inactivation of the Agr system does not alter the competitiveness of the mutants. Considering that in sterilized soil, cell density is higher than in biotic soil (about 4 log) and that scavenging of signal molecules is more limited, accumulation of signal molecules is expected. This suggests that, under these experimental conditions, the AgrA-controlled features may not be essential for growth.

**Table 2:** **Competitive Indexes of co-cultured listerial strains over a 14-days period in sterilized soil microcosms**.

**Time (Days)**	**Response-negative mutant/parental strain**	**Signal-negative mutant/parental strain**	**Response-negative mutant/signal-negative mutant**
0	1	1	1
2	1.91	3.21	3.74
4	1.50	1.37	1.03
7	1.81	1.13	1.63
14	1.67	1.68	1.12

^*^ Indicates when the CI significantly differed from the time 0 (repeated-measures ANOVA, Tukey, P < 0.05).

**Figure 4:**
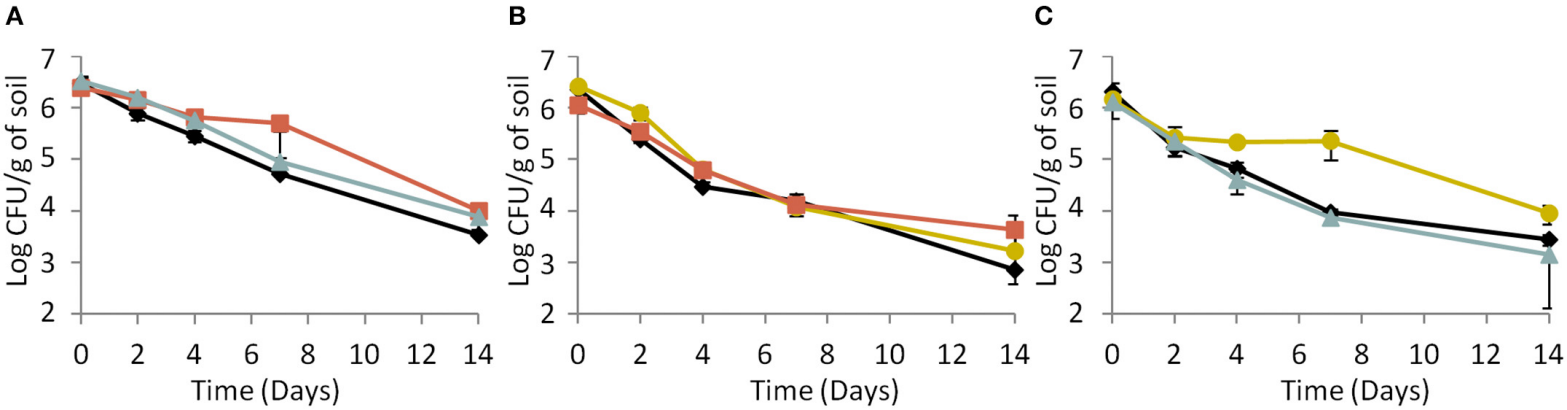
**Dynamics of (A) the parental strain, (B) the response-negative mutant and (C) the signal-negative mutant populations in biotic soil microcosms**. (♦) Single culture, (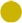) co-culture with the parental strain, (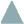) co-culture with the response-negative mutant, (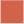) co-culture with the signal-negative mutant. Error bars represent the standard deviation from three replicate samples value.

Under biotic conditions, survival of the parental strain (Figure 4A) and the response-negative mutant (Figure 4B) did not vary whatever the co-culture tested. On the opposite, results indicated a significant (ANOVA, *P* < 0.05) improvement of the signal-negative mutant's survival when co-cultured with the parental strain but not when co-cultured with the response-negative mutant (Figure 4C). This indicates that the fitness of the signal-mute strain depended of the presence or absence of cells with active Agr systems and that the parental strain provided a benefit to this mutant. In addition to this, CI measurements showed that under biotic conditions, the CI of the response-negative mutant co-incubated with the parental strain significantly (ANOVA, *P* < 0.05) decreased over time (Table 3). Under these conditions, the parental strain had a significant competitive advantage over the response-negative mutant. The inability to respond to Agr communication was detrimental to the survival of the response-negative mutant. This is supporting the idea that the Agr communication system is important for competitiveness of *L. monocytogenes* in soil when complex microbial communities are active. When the signal-negative mutant and the parental strain were co-inoculated, the analysis of variance showed that the CI did not significantly vary over the 14 days of the experiment except after 2 days of incubation where the CI of the signal-negative mutant was significantly lower than the parental strain (*P* < 0.05) (Table 3). These results suggest that, at later stages of incubation, the fitness of the signal-negative mutant was similar to the fitness of the parental strain during co-culture, confirming that the presence of the parental strain improved competitiveness of the signal-negative mutant. Finally, when the two mutants were tested in biotic soil microcosms, the CI did not vary significantly over time (Table 3) meaning that none of the mutants took advantage over the other during the 14 days of incubation.

**Table 3:** **Competitive Indexes of co-cultured listerial strains over a 14-days period in biotic soil microcosms**.

**Time (Days)**	**Response-negative mutant/parental strain**	**Signal-negative mutant/parental strain**	**Response-negative mutant/signal-negative mutant**
0	1	1	1
2	0.54	0.31^*^	0.80
4	0.20^*^	0.31	0.50
7	0.25^*^	1.26	0.49
14	0.26^*^	1.74	1.95

* *Indicates when the CI significantly differed from the time 0 (repeated-measures ANOVA, Tukey, P < 0.05)*.

These results show first of all that signal molecules accumulate to levels sufficient to promote induction of the Agr communication system. The minimal threshold required to induce communication and cell-density-dependent gene expression depends on properties of the environment such as water availability, mass-transfer (Dulla and Lindow, 2008) and cell distribution (Hense et al., 2007). Under specific environmental conditions, with restricted diffusion of signal molecules, quorum can be reached even in small size populations. For example, on the surface of leaves, as few as 10 aggregated cells of *Pseudomonas syringae* can reach the quorum size (Dulla and Lindow, 2008). The local characteristics of soil such as the rates of diffusion and degradation of signal molecules and the cell density could generate a social environment propitious to communication between cells of *L. monocytogenes* even if present in small size populations.

Secondly, these results suggest that cells of the signal-mute population perceive and integrate signals produced by the parental strain into a concerted Agr response that restored the fitness of the signal-mute mutant. Such improvement was not observed with the response-negative mutant suggesting that under these experimental conditions, the Agr communication system regulates intracellular factors (private goods) rather than exo-products (public goods). Production of private goods promotes fitness advantage at the level of the individual cell in the bacterial models *Pseudomonas aeruginosa* and *Bacillus subtilis* (Dandekar et al., 2012; Darch et al., 2012; Oslizlo et al., 2014). Control of private goods by the Agr communication system is supported by results of transcriptomic analyses. Indeed, gene expression profile of the response-negative *ΔagrA* mutant indicated that deletion of *agrA* resulted in deregulations of amino acids, purine, and pyrimidine synthesis pathways and nitrogen transport (Garmyn et al., 2012). In soil, adaptation of *L. monocytogenes* requires an extensive reprofiling of gene expression (Piveteau et al., 2011) and genes coding proteins involved in cellular processes (transport proteins) and intermediary metabolism (specific pathways for metabolism of carbohydrates) including chitinases and β-glucosidases are upregulated. In the telluric environment where nutrients can be scarce, bacteria must be able to use a large range of carbon and nitrogen sources, for example cellulose and by-products of its hydrolysis (vegetal residues) and chitin (arthropod exoskeleton and cell wall of fungi) polymers largely represented in nature, and to synthesize specific enzymes for their catabolism. The ability of *L. monocytogenes* to acquire and use these energy sources could be critical for its saprophytic life in soil.

Recently, chitin hydrolysis by *L. monocytogenes* was reported to be under the control of the Agr System (Paspaliari et al., 2014). In our experiment, although chitin hydrolysis could generate public goods, we did not evidence any detrimental effect of the presence of mutants to the fitness of the parental strain. Agr mutants did not seem to act as cheaters exploiting the benefit of cooperation under our experimental conditions. In soil, cellular density may be locally inappropriate to gain benefit of cooperation. Others have shown that induction of private goods can be dominant and mask the benefits of public goods (Dandekar et al., 2012). Considering social traits are vulnerable to cheaters, in populations of *L. monocytogenes*, Agr mutants should be isolated from environments where Agr communication mediates social traits. However, at the moment, no environmental or clinical isolates of *L. monocytogenes* has been reported with mutations in *agrBDCA*. On the opposite, *P. aeruginosa* cheaters have been isolated from specific, confined environments where diffusion of signal molecules is low and where the pathogen is able to settle for a long period (Sandoz et al., 2007). Characteristics of the various environments where most isolates of *L. monocytogenes* have been collected so far are not propitious to the emergence of cheaters.

## Conclusion

The results reported here give new insights into the role of the Agr communication system in complex natural settings. First of all, the Agr communication system is required for optimal survival of *L. monocytogenes* in soil; secondly, it provides a benefit to *L. monocytogenes* populations in soil; thirdly, in the natural environment, production of signal molecules triggers a response in the receiving cells; and fourthly, the Agr system controls private goods. The question of whether the Agr system is a social trait of listerial populations remains to be investigated further. Indeed, the fact that the Agr system controls private goods does not exclude that it also controls public goods in specific habitats of *L. monocytogenes*.

### Conflict of interest statement

The authors declare that the research was conducted in the absence of any commercial or financial relationships that could be construed as a potential conflict of interest.
